# The potential for medicinal cannabis to help manage challenging behaviour in people with intellectual disability: A perspective review

**DOI:** 10.1177/02698811231209192

**Published:** 2023-11-08

**Authors:** Laura Korb, Samuel Tromans, Bhathika Perera, Nagina Khan, Lisa Burrows, Richard Laugharne, Angela Hassiotis, Victoria Allgar, Daryl Efron, Ian Maidment, Rohit Shankar

**Affiliations:** 1 Haringey Learning Disability Partnership, Barnet Enfield and Haringey Mental Health NHS Trust, London, UK; 2 Department of Population Health Sciences, University of Leicester, Leicester, UK; 3 Adult Learning Disability Service, Leicestershire Partnership NHS Trust, Leicester, UK; 4 North East London NHS Foundation Trust, London, UK; 5 Division of Psychiatry, University College London, London, UK; 6 Centre for Addiction and Mental Health, Toronto, Canada; 7 Royal Cornwall Hospitals NHS Trust, Truro, UK; 8 Cornwall Intellectual Disability Equitable Research (CIDER), University of Plymouth Peninsula School of Medicine, Truro, UK; 9 Peninsula Clinical Trials Unit, Faculty of Health, University of Plymouth, Truro, UK; 10 Royal Children’s Hospital, Melbourne, Australia; 11 Murdoch Children’s Research Institute, Melbourne, Australia; 12 Department of Paediatrics, University of Melbourne, Melbourne, Australia; 13 College of Life and Health Sciences, Aston School of Pharmacy, Aston University, Birmingham, UK

**Keywords:** Medicinal cannabis, developmental disorders, intellectual disability, challenging behaviour, polypharmacy

## Abstract

**Background:**

Around 2% of the population have intellectual disabilities. Over one-third people with intellectual disabilities (PwID) present with ‘challenging behaviour’, which nosologically and diagnostically is an abstract concept. Challenging behaviour is influenced by a range of bio-psycho-social factors in a population, which is unable to suitably comprehend and/or communicate concerns. This predisposes to poor health and social outcomes. There is no evidence-based treatments for managing challenging behaviour. Cannabidiol (CBD) and tetrahydrocannabinol (THC) are being trialled for a range of disorders, which are over-represented in PwID and provoke challenging behaviours, such as severe epilepsy, spasticity, post-traumatic stress disorder, social phobia, pain, etc.

**Methods:**

This perspective review explores the different conditions, which benefit from medicinal CBD/THC preparations, by analysing recent literature from neurobiological, pre-clinical and clinical studies related to the topic. The evidence is synthesised to build an argument of the therapeutic benefits and challenges of medicinal cannabis to manage severe challenging behaviour in PwID.

**Results:**

There is developing evidence of medicinal CBD/THC improving psychiatric and behavioural presentations in general. In particular, there is emergent proof in certain key areas of influence of medicinal CBD/THC positively supporting challenging behaviour, for example in children with neurodevelopmental disorders. However, there are significant challenges in employing such treatments in vulnerable populations such as PwID.

**Conclusion:**

Further clinical research for the considered use of medicinal CBD/THC for challenging behaviour management in PwID is needed. Strong co-production with experts with lived experience is needed for further testing to be done in this exciting new area.

## Intellectual disability and challenging behaviour

Disorder of intellectual development, popularly called intellectual disability (ID) (learning disability in the UK), is characterised by significant limitations in intellectual functioning and adaptive behaviour, impacting considerably on cognitive, conceptual, social and practical skills of everyday life, with the onset during the first 18 years of life (i.e. the developmental period; World Health Organization, 2018).

Around 2% of the population have ID (Maulik et al., 2011). People with intellectual disabilities (PwID) also often present with behaviour that causes a challenge to themselves, family members, carers and/or services (National Institute for Health and Care Excellence, 2015). These behaviours encompass a broad range of manifestations including, but not limited to, self-harm, physical aggression towards others, screaming, stereotypy, smearing or public exposure (National Institute for Health and Care Excellence, 2015). The individual’s level of ID and other characteristics, such as communication difficulties, including those relating to sensory impairment, play a significant role in the frequency and intensity of presenting challenging behaviours (Bowring et al., 2019; National Institute for Health and Care Excellence, 2015). Challenging behaviours occur in approximately 18% of PwID, although the prevalence varies according to multiple factors, such as the severity of ID, presence of frequently co-occurring conditions such as autism (Weiss, 2003) and attention-deficit hyperactivity disorder (ADHD) and the environment, with a heightened prevalence in inpatient settings (Bowring et al., 2019).

A range of biopsychosocial factors influence challenging behaviour in PwID. Common physical causes include constipation, pain (e.g., dental, gynaecological), sleep disturbances, infections, as well as chronic conditions known to commonly co-occur with ID, such as epilepsy (Bowring et al., 2019). Additionally, prevalence rates of major mental illness in PwID is estimated to be 3–5 times greater than that of the general population (Walton et al., 2022). Life events, such as bereavement and other environmental fluctuations (carer change, housing change, etc.), are some of the other factors that can increase anxiety presenting as challenging behaviour in this vulnerable population (National Institute for Health and Care Excellence, 2015).

There is evidence that challenging behaviours are more common in certain genetic syndromes. Lesch–Nyhan, Angelman, Fragile X, Cri du Chat, Smith–Magenis, Prader-Willi and Cornelia de Lange syndromes are associated with significantly higher levels of aggression compared to other genetic disorders, such as Williams and Down syndromes (Powis & Oliver, 2014).

## Current management of challenging behaviour in PwID

Challenging behaviours are the most common reason for referral to healthcare services in PwID, with significant numbers being prescribed psychotropic medication without a diagnosed mental disorder (McMahon et al., 2020).

Various psychotropic and psychosocial interventions are used to support PwID and manage challenging behaviours (National Institute for Health and Care Excellence, 2015). Current practice recommendations are largely based on expert opinions and small-scale studies. Recommended interventions for challenging behaviour without an underlying mental illness are non-pharmacological and involve positive behaviour support (PBS) (Gore et al., 2022). However, the use of PBS is only supported by low-grade evidence. In 2020, a randomised controlled trial investigating PBS strategies for PwID and challenging behaviour did not show any evidence for effectiveness (Strydom et al., 2020).

The National Institute for Health and Care Excellence (NICE) includes describes appropriate and short-term use of antipsychotic medications, in cases where other interventions have failed, where there is significant associated risk and/or co-occurring mental illness (National Institute for Health and Care Excellence, 2015). Nevertheless, the evidence base for using antipsychotic medications to treat challenging behaviour is limited. A randomised controlled trial comparing the use of risperidone, haloperidol and placebo in the treatment of aggressive challenging behaviour in patients with ID found no evidence of a worsening response in those treated with placebo in comparison to either antipsychotic (Tyrer et al., 2008). Alongside the uncertainty surrounding the effectiveness of antipsychotics for treating behavioural challenges in PwID, there are substantial concerns about the increased sensitivity to certain side effects within this population and the tendency to over-rely on antipsychotics in cases with no diagnosed mental illness (Sheehan et al., 2017a, 2017b). There is a need for caution in relation to prescribing for PwID, as individuals with significant communication difficulties may be less able to describe their subjective experiences, leading to a risk of adverse reactions remaining potentially undetected (Ward et al., 2013).

Clinically, it is not uncommon that the combination of psychosocial and pharmacological interventions is ineffective in managing challenging behaviour in PwID. This can in some cases lead to restrictive practices, as the priority becomes keeping PwID safe, in addition to protecting other persons (Bowring et al., 2019; The National Institute for Health and Care Excellence, 2015).

## Medicinal cannabis

Medicinal cannabis is either derived from the cannabis plant, or synthetically produced, to mimic the components of medicinal cannabis (Freeman et al., 2019). The two main chemicals within the cannabis plant (cannabinoids) are delta-9- tetrahydrocannabinol (THC) and cannabidiol (CBD). THC is a psychoactive component of cannabis, and its therapeutic effects include reduction in nausea and vomiting (Gonzalez-Rosales & Walsh, 1997). CBD is a non-psychoactive chemical, exhibiting multiple therapeutic effects, including having anti-epileptic, anxiolytic and analgesic properties (Peng et al., 2022).

NICE recommendations for medicinal cannabis (The National Institute for Health and Care Excellence, 2021) include use for chemotherapy-induced nausea and vomiting, spasticity secondary to multiple sclerosis following failure to respond to other drugs and certain severe treatment-resistant types of epilepsy. The current UK-licenced preparations and their indicated uses are in Table 1.

**Table 1.** Comparison of current licenced and unlicenced medicinal cannabis products available in the UK.

Name	Form	Composition	Licenced indication	Possible indication for PwID
Epidyolex (Electronic medicines compendium, 2022)	Liquid	CBD 100 mg/ml	Seizures in children (aged two or older) with two rare forms of epilepsy, Lennox–Gastaut syndrome and Dravet syndrome, in conjunction with clobazam, and as adjunctive therapy of seizures associated with tuberous sclerosis complex	For persons with ID with co-occurring epilepsy, with uncontrolled seizures contributing to the challenging behaviour (e.g. during peri-ictal period), as well as severe anxiety and challenging behaviour
Sativex (Electronic Medicines Compendium, 2022)	Oromucosal spray	Each ml contains: 27 mg THC and 25 mg CBD.	Multiple sclerosis associated spasticity unresponsive to other treatments	This medication has been found to be effective to treat tic disorder (Koppel, 2015). Tics are another common co-occurring condition with neurodevelopmental conditions
Nabilone (Electronic Medicines Compendium, 2021)	Capsule	Synthetic cannabinoid (mimics effect of THC)	Chemotherapy-induced nausea and vomiting unresponsive to conventional anti-emetics	No clear indication
CBD:THC products	Drops (oil) or inhaled (dried flower)	CBD and THC titrated up as per dosing protocol of the prescribing clinic	No current licensed indications. These forms are prescribed in private practice (off-label and off-licenced use)	There is real-world observational data and emerging evidence suggesting improvements in several mental health conditions associated with challenging behaviour including anxiety, post-traumatic stress disorder (PTSD), autism and ADHD
Over the counter and internet CBD products (Chesney et al., 2020)	Drops (oil), gummies, capsules, spray	Often marketed as ‘pure CBD’ but could contain low amounts of CBD or some THC, as products are not regulated	No current licenced indications. Health stores claim that CBD products have a range of indications including pain relief, anxiety insomnia, PTSD related symptoms, depression and lowering blood pressure	Not advisable given the lack of control over the dosing and lack of medical guidance

## Medicinal cannabis and psychiatric conditions

The Royal College of Psychiatrists (2019) published a statement on cannabis-based medicinal products, highlighting the requirement for high-quality research in this field.

Studies have emerged to suggest the use of prescribed cannabis-based medicinal products for a range of mental disorders, including social anxiety, psychosis, insomnia and PTSD (Sarris et al., 2020). In a cohort study assessing the clinical outcomes of patients with generalised anxiety disorder who were prescribed cannabis products (including oils/dry flower or a combination thereof), a clinically significant improvement in anxiety was reported, with a corresponding acceptable safety profile (Rifkin-Zybutz et al., 2023). As highlighted earlier, these mental disorders are overrepresented in PwID. Table 2 shows a summary of the studies evaluating the efficacy and the different medical cannabis preparations for mental health and neurodevelopmental conditions discussed in this article.

**Table 2.** A summary of the studies evaluating the efficacy of medical cannabis preparations for mental health and neurodevelopmental conditions.

Study	CBD preparation	Study design	Study population	Findings
Bar-Lev Schleider et al. (2019)	Most patients: Cannabis oil with a THC:CBD ratio of 1:20 (30% CBD, 1.5% THC) Participants with a history of sensitivity to prior medications: Cannabis oil with a THC: CBD ratio of 1:20 (15% CBD, 0.75% THC) Participants with severe sleep disturbance or significant aggressive behaviour: addition of 3% THC oil following initial treatment phase	Prospective cohort study (Outcome measurement at 6 months)	Autistic children and adolescents (*n* *=* 188)	Following 6 months of treatment, 60% of participants were assessed (*n* *=* 93), of which 28 patients (30.1%) reported a significant improvement, 50 (53.7%) moderate, 6 (6.4%) slight and 8 (8.6%) had no change in their condition’ (Bar-Lev Schleider et al., 2019) Additionally, 28 patients (30%) reported adverse effects, including restlessness
Cooper et al. (2017)	Sativex oromucosal spray (1:1 ratio; delta-9-tetrahydrocannabinol to cannabidiol)	Double-blind randomised placebo-controlled trial (Outcome measurement at day 42)	Adults with ADHD (*n* = 30)	No significant difference from placebo with respect to cognitive performance and activity level, inattention or emotional lability. Nominally significant improvements in hyperactivity/impulsivity (*p* *=* 0.03) and inhibition (*p* = 0.05), though these findings did not reach the threshold for significance after adjustment for multiple testing
Rifkin-Zybutz et al. (2023)	Variable cannabis-based medical products	Cohort study (Outcome measurement at 1, 3 and 6 months)	Patients with generalised anxiety disorders (*n* = 302)	Significant improvements in anxiety, sleep quality and quality of life at all three time points were reported (*p* *<* 0.001)
Sarris et al. (2020)	Variable preparations used in constituent studies	Systematic review	Patients with major psychiatric disorders (13 eligible studies)	Depression: No benefit from delta-9-tetrahydrocannabinol Mania: No benefit from CBD PTSD: Weak evidence from case studies Schizophrenia: Mixed evidence for use as an adjunctive treatment Social anxiety: Tentative support for CBD Sleep disorders: Weak evidence from case studies
Silva Junior et al. (2021)	Variable preparations used in constituent studies	Systematic review	Autistic patients (nine eligible studies)	Some eligible studies reported reductions in the frequency and/or intensity of various symptoms, ‘including hyperactivity, attacks of self-mutilation and anger, sleep problems, anxiety, restlessness, psychomotor agitation, irritability, aggressiveness, perseverance and depression’, as well as improvements in ‘cognition, sensory sensitivity, attention, social interaction, and language’ (Silva Junior et al., 2021). Adverse effects included ‘sleep disorders, restlessness, nervousness and change in appetite’ (Silva Junior et al., 2021).
Timler et al. (2023)	‘A mix of THC and CBD (3:2 ratio; 25 mg/ml THC; 17 mg/ml CBD)’. (Timler et al., 2023)	Double-blind randomised placebo-controlled crossover trial (Outcome measurement at 18 weeks)	Adults diagnosed with dementia based in residential care facilities (*n* *=* 21)	No significant difference from placebo with respect to behaviour, quality of life and pain. However, a significant difference was found with respect to agitation, in favour of the treatment group

## Medicinal cannabis and seizures

CBD is now licenced for treatment-resistant seizures in two epileptic encephalopathies significantly associated with ID, Lennox–Gastaut and Dravet’s syndrome (The National Institute for Health and Care Excellence, 2022). Over 90% of patients with the Lennox–Gastaut syndrome have intellectual impairment (Camfield & Camfield, 2002), frequently with co-occurring autistic features, psychosis, hyperactivity and behavioural problems. Similarly, the Dravet syndrome is associated with nearly 90% having cognitive impairment of whom two-thirds have IQ scores less than 50 (Jansson et al., 2020). In Dravet’s syndrome, many patients are considered to have significant behavioural problems with behavioural disturbances being a strong predictor of quality of life.

While the impact of CBD on seizures is positive, there has been limited evaluation of its impact on non-seizure outcomes, particularly challenging behaviour. Parents of children given various oral cannabis extracts for epilepsy have reported improvements in mood, behaviour and alertness (Press et al., 2015) Importantly CBD has been shown to be generally well-tolerated.

## Medicinal cannabis and neurodevelopmental and neuropsychiatric conditions

There has been increasing interest in the use of medicinal cannabis in other neurodevelopmental disorders that commonly co-occur with ID, particularly autism and ADHD. A prospective cohort study of medical cannabis treatment programme of 188 autistic children found cannabis oil containing 30% CBD and 1.5% THC to be a well-tolerated, safe and effective option to relieve symptoms, particularly seizures, tics, depression, restlessness and rage attacks (Bar-Lev Schleider et al., 2019). An open-label study of 110 autistic children and adolescents treated with a CBD-rich preparation reported significant improvements, particularly in social communication. A systematic review on varied cannabis-based products used in autistic people, reported improvement in autism-related symptoms, including self-mutilation, anger, hyperactivity, sleep problems, anxiety, irritability, aggressiveness and restlessness (Silva Junior et al., 2021). A review of evidence compared the safety and efficacy of commonly used medications in autism with different preparations of commonly available medicinal cannabis. While the lack of placebo-controlled trials of any form of medicinal cannabis for autistic persons was recognised, lower-level evidence indicated that a preparation of 20:1 CBD:THC cannabis product may be effective for symptoms such as aggression and was generally well-tolerated and safe (Holdman et al., 2022).

Results were more modest from a single-centre, randomised controlled double-blind study using Sativex (a nasal spray containing THC and CBD) on 30 people with ADHD (Cooper et al., 2017). No significant differences were found between the active treatment group and the placebo group in cognitive performance or activity level; the active treatment group showed improvements in hyperactivity, inhibition and inattention compared to the placebo group, though this difference was not statistically significant after adjustment for multiple testing.

There is also emerging evidence that CBD- and THC-based medical products may be a potential option for patients with behavioural and psychological symptoms associated with dementia. A small, randomised placebo-control crossover trial of people living in residential care facility in Australia with a diagnosis of dementia indicated a significant reduction in agitation in those treated with a mix of THC and CBD (3:2 ratio) compared with placebo (Timler et al., 2023). They reported that the medicinal products used were safe and well-tolerated, as well as receiving positive qualitative feedback from both the families of the trial participants as well as staff, specifically regarding sleep and relaxation.

## Why consider CBD/THC in PwID and challenging behaviour?

There is a lack of high-level clinical evidence regarding the specific use of CBD/THC to treat challenging behaviour in PwID. This is understandable, as challenging behaviour covers a wide range of behaviours with a varied aetiology. However, many of these potential aetiologies individually are being shown to benefit from preparations involving CBD/THC. The examples outlined of the developing evidence in seizure-related syndromes strongly linked to ID and behavioural problems, autism, spasticity and mental illness, lend weight to argue for a serious consideration of CBD/THC as a treatment option for this vulnerable cohort. It could also be argued that CBD/THC might provide an opportunity to rationalise the current high levels of inappropriate psychotropic prescribing, which has been linked to significant iatrogenic harm and risk of premature mortality (Branford & Shankar, 2022). Additionally, it is thought that as polypharmacy occurs in 38% of PwID, McMahon et al. (2020) prescribed cannabis-based products could be the ‘lesser of two evils’ in management of behaviours that challenge by helping optimise medication.

There is emerging evidence that CBD may help reduce severe behaviour problem in children and adolescents with ID. A small pilot study (*n* = 8) indicated that CBD may be safe and effective for this patient group (Efron et al., 2021). However, further research is needed, and there is a current large randomised placebo-controlled trial in children and adolescents with ID (Efron et al., 2020).

## Possible pharmacological mechanism

The endocannabinoid system has a key role in behaviour and neurodevelopment (Basavarajappa et al., 2009; Efron et al., 2020). The mode of action for medicinal cannabis in mood disorders and behavioural issues is not clear, but possible mechanisms include changes in neurotransmission and calcium homeo-stasis, and anti-inflammatory and/or anti-oxidant effects (Campbell et al., 2017; Poleg et al., 2019). The anti-inflammatory, anti-oxidant and neuroprotective properties of CBD provide a possible mechanism for treating behavioural problems in young people with developmental disorders (Efron, 2021; Poleg et al., 2019). CBD may possess anxiolytic activity via neurotransmitter systems linked to the regulation of anxiety including the serotonin 5-HT_1A_ receptor, the cannabinoid type one receptor (CB_1_R) and the transient receptor potential vanilloid type 1 (TRPV1) receptor (Blessing et al., 2015).

## Practical challenges in researching the utility of medicinal cannabis in PwID and challenging behaviour

### Concerns about adverse effects

As for all prescribed medicines, medical cannabis carries the possibility of adverse effects. A systematic review found that the most common adverse event was dizziness in short-term use and that there were few high-quality trials of long-term medical cannabis use (Wang et al., 2008). In his review, Arnold (2021) reported that cannabis is not associated with fatal overdose, with THC having a wide safety margin that is 750 times greater than the typical intoxicating dose. However, it is important to note that higher doses of THC may lead to anxiety, panic, tachycardia, and in some cases, subtle cognitive deficits such as short-term, reversible memory impairment. There is a known risk of inducing a psychotic episode with THC use, but this was described as rarely occurring when cannabis is the only drug used. Comparatively, CBD was found to be well tolerated even at extremely high doses, with the main side effect being diarrhoea, and the main concern being related to potential drug interactions as CBD inhibits the activity of CYP450 enzymes, which as discussed previously, can affect the metabolism of certain medications (Arnold, 2021).

Another significant concern is regarding the potential for dependence and withdrawal syndromes. A review paper found that the risk of recreational cannabis dependence occurs more commonly in those using high THC strains with daily heavy use from an early age (Curran et al., 2016). There is a lack of research into the risk of dependence for prescribed medical cannabis, though there are recommendations for reducing the risk of dependence based on available evidence, including the importance of considering the ratio of THC to CBD, avoidance of black market products, close monitoring by a health professional, lower daily use and an individualised person-centred plan that includes weighing up the risks of using alternative medications (Schlag et al., 2021).

With respect to interactions with other medicine, raised liver enzymes can occur in patients taking concomitant sodium valproate, particularly in the initial months of treatment. In clinical trials, the incidence of alanine aminotransferase elevations (three times the upper normal limit) was 12% in patients receiving CBD treatment compared to less than 1% in patients on placebo. Elevated levels of aspartate aminotransferase, dizziness, weight loss, sedation and gastrointestinal disorders (such as nausea and vomiting) have also been reported (Electronic medicines compendium, 2022). Furthermore, CBD can interact with other medications, principally related to pharmacokinetic effects involving CYP450 isoenzymes (Lopera et al., 2022), including Clobazam (increasing Clobazam metabolite levels and potentially increasing sedative effects) and Phenytoin (increasing Phenytoin levels) (Electronic medicines compendium, 2022).

### Research concerns

PwID are often excluded in research groups for a variety of reasons, including concerns about consent and vulnerability (Sellers et al., 2022). Due to the lack of a robust evidence base, there also remains the question over the both the benefits and risks of possible unknown harms of using cannabis-based medicines. This can hinder the development of an evidence base for this specific group of individuals, as involving a vulnerable population who cannot consent in a pharmaceutical trial is challenging. However, not developing an evidence base specifically for PwID could deprive them of a treatment that could potentially improve their lives (Alexander et al., 2021).

### Population challenges

PwID are a heterogeneous group, with considerable variation existing in this population in relation to aetiology, severity and comorbidity profile. Those with mild ID represent 85% of PwID. Their needs are much more driven by a lack of service flexibility and social inclusion. Those with moderate to profound ID, representing 15% of PwID, are defined largely by significantly greater health and social needs. On average, PwID tend to have 11 chronic health conditions (Kinnear et al., 2018). The prevalence of co-occurring conditions and polypharmacy increase proportionate to the severity of ID.

An increased frequency and intensity of challenging behaviours are seen in those with more severe ID, who are also likely to have greater communication deficits (Bowring et al., 2019). Furthermore, this patient group is more likely to lack mental capacity to provide informed consent to treatment, and any corresponding best interests decision needs to be thoroughly considered, with input from family members, carers and other members of the multidisciplinary team as outlined in the Mental Capacity Act 2005 in the UK or similar guidance in other countries. The emergence of challenging behaviour is thus dependent on not only the diverse aetiologies and differing needs but the individual and his/her level of disability and associated co-occurring conditions.

### Defining challenging behaviour and the influence of diagnostic overshadowing

It is important that non-cause-specific treatment for challenging behaviours is only considered when no obvious remediable cause is found. Too often PwID are being ‘diagnostically overshadowed’. This happens when an assumption is made of the challenging behaviour being a part of the disability, without exploring other factors, such as biological, environmental/social and psychological determinants. This has led to the over-prescribing of psychotropic medications. Thus, medicinal cannabis should neither become a proxy for treatable medical and psychological conditions nor replace evident social and environmental support gaps. The nature and frequency of challenging behaviour needs to be taken into account. What amounts to ‘serious’ challenging behaviour needs to be defined to inform trials of medicinal cannabis. Thus, any developments to involve medicinal cannabis prescribing requires sound safeguards to assure mitigation of diagnostic overshadowing, possibly by establishing a clear pathway or decision support tool.

## Co-production

An important aspect of any research in PwID needs to be meaningful co-production with people with lived experience of the condition. This needs to include the voices and opinions of those with ID and challenging behaviour and also provide opportunities for participation for their families and carers. Given the range and heterogeneity of aetiology for the presentation, serious thought needs to be given to make co-production representative and meaningful. Issues of ethics and consent (or the lack of it) are significant for this vulnerable population.

## Conclusion

There remains a lack of effective psychosocial and pharmacological interventions for managing serious challenging behaviour in PwID, which has significant implications on their quality of life, health and social outcomes. Too often, the research community has not been bold enough to insist that research is vital for the care of PwID. Using the complex issue of capacity to consent as an excuse not to progress research is simply not good enough. There is therefore the need to look for alternative, safer treatment approaches, principally to be used in combination with existing non-pharmacological management options. Medicinal cannabis particularly CBD appears to offer a potentially exciting new development, based on its reported therapeutic benefits for PwID in treating generalised anxiety disorder, as well as behavioural and psychological symptoms associated with dementia. Furthermore, while a risk of adverse effects certainly exists, these studies report reassuring findings with respect to the safety profiles of these medicines (Rifkin-Zybutz et al., 2023; Timler et al., 2023). Nevertheless, further research is required to determine the clinical utility of medicinal cannabis, specifically in relation to treatment of challenging behaviour in PwID. Given the possible clinical implications and potential benefits, consideration should be given to undertaking robust, well-powered prospective studies into medicinal cannabis in individuals with PwID and challenging behaviour.
